# Modeling of Motion Characteristics and Performance Analysis of an Ultra-Precision Piezoelectric Inchworm Motor

**DOI:** 10.3390/ma13183976

**Published:** 2020-09-08

**Authors:** Bo Zhao, Ri Fang, Weijia Shi

**Affiliations:** The Institute of Ultra-Precision Optoelectronic Instrument Engineering, School of Instrumentation Science and Engineering, Harbin Institute of Technology, Harbin 150001, China; hitzhaobo@hit.edu.cn (B.Z.); Ri_FANG@163.COM (R.F.)

**Keywords:** piezoelectric inchworm motor, multi-physics field coupling simulation, motion gesture, driving force, travel

## Abstract

Ultra-precision piezoelectric inchworm motor (PIM) is widely used in the optical equipment, microelectronics semiconductor industry and precision manufacturing for motion and positioning, but the multi-physics field simulation model for estimating PIM performance and assisting motor design is rarely studied. The simulation model in this paper aimed to provide researchers with direct and convenient PIM performance evaluation to assist the motor design and development. According to the existing advanced inchworm motor products, a multi-physics field coupling model involving solid mechanics and electrostatics using the finite element method (FEM) was established. The motion gesture and performance (driving force and travel) of the PIM were analyzed, respectively. The simulation results showed that the motion gesture of the inchworm motor was well consistent with that of the actual motor product. The driving force from the simulation was close to that of the actual product, and the maximum error was 2.8%. As for the PIM travel, there was a maximum travel error of 0.6 μm between the simulation and official data. The performance parameters of the piezoelectric materials under certain specifications can be simulated by the multi-physics field coupling model. Therefore, the multi-physics field coupling simulation model is suitable for PIM performance evaluation and assisting motor development.

## 1. Introduction

A piezoelectric motor is common in ultra-precision motion, positioning, and micro-assembly with its characteristics of high resolution, instant response, and long travel [1,2,3,4]. It supports application scenarios such as the fine tuning of the mirror array of radio telescopes [5], micro adjustments in ultra-precision optical equipment [6], and gesture control of engineering robots [7]. For a piezoelectric motor with an inchworm’s walking mode, it is called a PIM. The high-load and ultra-compact PIM has great potential in the field of demanding semiconductor lithography, because it has high requirements for reliability, position resolution, and long-term stability [8]. In particular, the PIM is very likely to be an advanced solution of a chunk motion actuator, replacing the conventional voice coil linear motor in the current lithography chunk.

**Figure 1:**
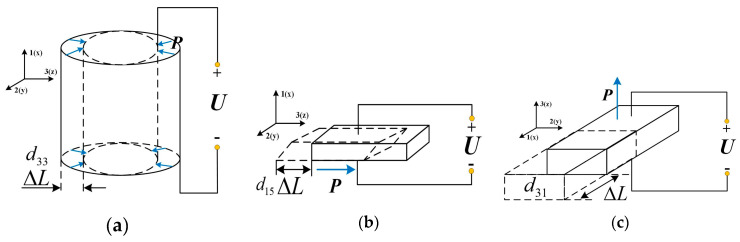
Strain modes of piezoelectric ceramics with three geometric structures: (**a**) *d*_33_ mode in a hollow cylinder; (**b**) *d*_15_ mode on a shear plane; (**c**) *d*_31_ mode on a long plane.

The working principle of a piezoelectric motor is an inverse piezoelectric effect. That is, when the voltage is applied to an unconstrained piezoelectric material (PM), the PM will produce geometric deformation [9]. The deformation movement is determined by the geometry of the PM, the direction of the electric field, and polarization. For example, the deformation modes of three common piezoelectric ceramics are shown in Figure 1. After polarization, only three piezoelectric strain modes—*d*_33_, *d*_15_, and *d*_31_ exist. For the *d*_33_ mode, the deformation direction is consistent with that of the electric field and polarization as shown in Figure 1a; the cuboid structure can also generate the *d*_33_ strain mode. For the *d*_15_ mode, if the electric field direction is on the *x*-axis of the global coordinate system and the polarization direction is perpendicular to it, the volume deformation (shear deformation) is the vector sum of the PM’s deformation on the *x*-axis and the *z*-axis as presented in Figure 1b; the deformation exists only on the *x*-axis and *z*-axis but not on the *y*-axis. For the *d*_31_ mode, illustrated in Figure 1c, if the electric field direction is on the *z*-axis, the deformation direction is on the *x*-axis. The motion gesture of the inchworm motor in this paper is composed of the *d*_33_ and *d*_15_ modes [10]. The *d*_33_ strain mode is responsible for the extension and contraction of the actuation legs in the vertical direction, while the *d*_15_ strain mode is responsible for generating shear deformation in the horizontal direction, which makes the motor shaft move in the same direction. This direct contact method without a flexible mechanism avoids the mechanical coupling process, so there is no reduction in accuracy and reliability [11].

The key technique of the ultra-precision piezoelectric motor has been widely studied. Simulation modeling is a powerful tool for PIM design in many studies. Li et al. [11] simulated the flexure clamp mechanism + actuator-type PIM using ANSYS FEM to perform stress analysis, but the mechanical coupling process of the PM deformation leading to the movement of the flexure clamp was not involved in the frequency simulation analysis. An ANSYS FEM simulation of a flexure clamping mechanism + middle-drive mechanism-type PIM was conducted by Ma et al. [12], and the related simulation also did not reflect the energy coupling process between the PM and clamping mechanism. Therefore, the common strategy between Li et al. [11] and Ma et al. [12] was to separately simulate the compliant mechanism and the driving mechanism (PM) of the system to indirectly obtain the desired output. As for PIM research in recent years, a four-foot rotary piezoelectric motor without a clamping mechanism was developed in Liu’s study [13], its transient simulation work took into account friction between the rotor and driving foot, but the key deformation mode of the PM and the abovementioned coupling problem were not reflected in the simulation. For the flexure mechanism + drive mechanism-type PIM research, such as Ma et al.’s [12], Zhang et al. [14] conducted stress and frequency analyses of the driving mechanism presented in a steady state simulation. To sum up the simulations in the above studies, the strain modes of the PM were not involved, the motion gesture of the PIM must be achieved by the correct strain mode of the PM. In addition, the mechanical coupling between the PM and flexure mechanism or motor shaft was rarely reflected. Finally, the travel performance of the PIM was obtained by actual measurement or indirect formula calculation; it was not capable of being obtained directly from the simulation model [12,13,14]. However, for the motor design, the relationship between the specifications of the PM (i.e., type and size) and precision motor performance (i.e., driving force and travel) is what professional developers want to know most. The above cases also appear in References [15,16,17,18,19]. In view of the issues mentioned, a multi-physics field coupling simulation involving electrostatics and solid mechanics using FEM was established for direct PIM performance evaluation to assist the motor design and development under certain PM.

In this paper, according to the advanced inchworm motor, which is developed by PI Motion, the multi-physics coupling simulation was built for PIM performance estimation. During movement, contact friction will occur between the actuation foot and the motor shaft. The contact friction is a highly nonlinear process which undoubtedly increases the complexity of the model and makes the model very difficult to solve. Therefore, the two studies were carried out as follows: (1) focusing on the analysis of the gesture motion of the four-foot PIM, a coupling model without a drive shaft was established; (2) a model with a driving shaft was established to study the motor’s performance.

## 2. Material

**Figure 2:**
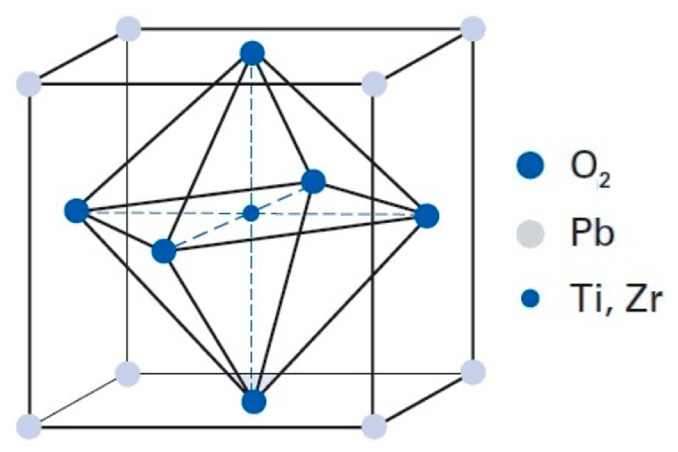
Tetragonal unit cell of soft piezo ceramic.

**Figure 3:**
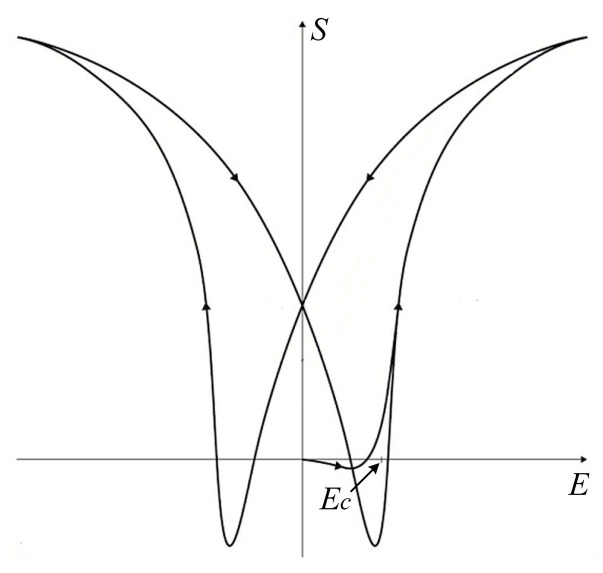
The ideal strain–electric field characteristic of soft piezo ceramic.

The core piezoelectric material of the PIM is PIC255 which is classification in accordance with EN 50324-1. The PIC255 belongs to “soft” ferroelectric ceramic based on lead zirconate titanate (PZT) and barium titanate. The tetragonal unit cell of “soft” piezo ceramic is shown in Figure 2. The “soft” ideal characteristic of general piezo ceramics can be explained by Figure 3. Figure 3 illustrates the strain–electric field characteristic of “soft” piezo ceramic when a bipolar voltage is applied. When the negative electric field increases to a critical positive electric field, *E_C_*, the strain gradually decreases. The strain increases with the increasing electric field when the electric field strength exceeds the *E_C_*. If the positive electric field decreases to critical negative electric field *−E_C_*, the strain increases gradually. When the electric field is less than *−E_C_*, the strain increases with the decreasing electric field. So PIC255 is applied to the actuator’s application in dynamic operations (alternating voltage). In addition, the Curie temperature of the PIC255 is 350 °C; it ensures that the PIC255 has extraordinary stability at normal temperature [20].

## 3. Simulation

**Figure 4:**
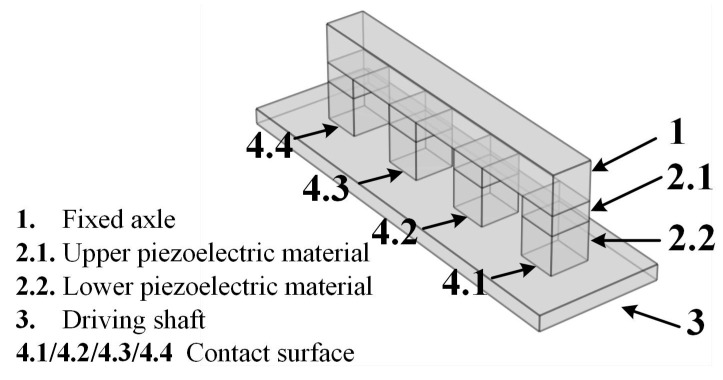
The simulation model of the inchworm motor.

According to PICA Shear actuation and PiezoWalker Motors of PI Motion in References [21,22], the PIM model was built as shown in Figure 4. The system consisted of a fixed axle (domain 1), four actuation legs, and a driving shaft (domain 3). Each actuation leg was composed of upper and lower piezoelectric materials (domain 2.1 and 2.2). In addition, it was necessary to remove the part of the driving shaft in the model when only paying attention to the gesture analysis of the four actuation legs. The three actuator products, P-123.01, P-143.01, and P-153.01, were simulated in this paper [23]. The simulation model was built using COMSOL FEM 5.5 software.

### 3.1. Physical Field Analysis

The whole physical model was the coupling of solid mechanics and electrostatics; the model with the driving shaft also involved the highly nonlinear contact friction between the four actuation legs and the driving shaft. Nonlinearity will sharply increase the difficulty of model solving, so the model needs to reduce the number of contact surfaces as much as possible. The minimum number of contact surfaces of the model with a drive shaft is four as presented in Figure 4. This model reduced the nonlinear contact surface as much as possible on the premise of ensuring the integrity of the motion process. As for the model without the driving shaft, the model solution can be easily realized due to the lack of contact friction.

#### 3.1.1. Solid Mechanics

The whole model including domain 1, domain 2.1 and domain 2.2, and domain 3 is described by solid mechanics. The two types of piezoelectric constitutive equations in solid mechanics were used to analyze the inverse piezoelectric effect which reflected the coupling between the structural (strain and stress) and electrical domains (the electric field). The strain and stress of the material can be obtained from the piezoelectric constitutive equations. The strain and stress of the volume elements on the contact surfaces produce normal and tangential force which is needed in the Coulomb friction model. Finally, the piezoelectric constitutive equation must be applied to all PM regions (domains 2.1 and 2.2).

The strain–charge constitutive equation reflects the coupling relationship between material stress and relative permittivity at constant stress which is shown in Equation (1):

(1) $$\begin{array}{l} S=s_{E}T+d^{t}E\\ D=dT+\varepsilon_{0}\varepsilon_{rT}E \end{array}$$

where *S* is the 6 × 1 strain matrix, reflecting the deformation degree of PM. *S_E_* is the sixth order flexibility coefficient square matrix, *T* is the 6 × 1 stress matrix, *d* is the 3 × 6 piezoelectric strain constant matrix, *E* is the 3 × 1 electric field strength matrix in the *X*, *Y*, and *Z* direction of the orthogonal global Cartesian coordinate, *D* is the electric displacement, *ε*_0_ is the vacuum dielectric constant, *ε_rT_* is the relative dielectric constant at constant stress, and *t* is the transposition symbol. The above coefficient matrix forms are determined by the PIC255 material characteristics. The PIC255 belongs to a tetragonal crystal system, so the flexibility coefficient matrix *S_E_* is the symmetrical matrix; it only contains six independent elements as shown in Equation (2):

(2) $$s_{E}=\left[\begin{array}{cccccc} s_{E11} & s_{E12} & s_{E13} & 0 & 0 & 0\\ s_{E12} & s_{E11} & s_{E13} & 0 & 0 & 0\\ s_{E13} & s_{E13} & s_{E33} & 0 & 0 & 0\\ 0 & 0 & 0 & s_{E44} & 0 & 0\\ 0 & 0 & 0 & 0 & s_{E44} & 0\\ 0 & 0 & 0 & 0 & 0 & s_{E66} \end{array}\right]$$

Generally, only three piezoelectric strain constants of the *d*_33_, *d*_15_, and *d*_31_ are left after polarization of PM, and its piezoelectric strain coefficient matrix becomes:

(3) $$d=\left[\begin{array}{cccccc} 0 & 0 & 0 & 0 & d_{15} & 0\\ 0 & 0 & 0 & d_{15} & 0 & 0\\ d_{31} & d_{31} & d_{33} & 0 & 0 & 0 \end{array}\right]$$

After PM polarization, the relative permittivity mainly exists in the directions that are both parallel (*ε_rT_*_33_) and perpendicular (*ε_rT_*_11_) to the polarization direction. The matrix form of the relative permittivity *ε_rT_* is as follows:

(4) $$\varepsilon_{rT}=\left[\begin{array}{ccc} \varepsilon_{rT11} & 0 & 0\\ 0 & 0 & 0\\ 0 & 0 & \varepsilon_{rT33} \end{array}\right]$$

The stress–charge constitutive equation reflects the coupling relationship between material strain and relative permittivity at constant strain. Equation (5) is as below:

(5) $$\begin{array}{l} T=c_{E}S-e^{t}E\\ D=eS+\varepsilon_{0}\varepsilon_{rS}E \end{array}$$

where *c_E_* is the sixth order stiffness coefficient square matrix, *e* is the 3 × 6 piezoelectric stress constant matrix, also called coupling matrix, and *ε_rS_* is the relative dielectric constant at constant strain.

The form of the stiffness coefficient matrix *c_E_* is the same as that of the flexibility coefficient matrix *S_E_*, which is also determined by the PIC 255 crystal system type as presented in Equation (6):

(6) $$c_{E}=\left[\begin{array}{cccccc} c_{E11} & c_{E12} & c_{E13} & 0 & 0 & 0\\ c_{E12} & c_{E11} & c_{E13} & 0 & 0 & 0\\ c_{E13} & c_{E13} & c_{E33} & 0 & 0 & 0\\ 0 & 0 & 0 & c_{E44} & 0 & 0\\ 0 & 0 & 0 & 0 & c_{E44} & 0\\ 0 & 0 & 0 & 0 & 0 & c_{E66} \end{array}\right]$$

Similarly, the form of piezoelectric stress coefficient matrix *e* strictly follows the form of strain coefficient matrix *d*; it is listed below:

(7) $$e=\left[\begin{array}{cccccc} 0 & 0 & 0 & 0 & e_{15} & 0\\ 0 & 0 & 0 & e_{15} & 0 & 0\\ e_{31} & e_{31} & e_{33} & 0 & 0 & 0 \end{array}\right]$$

This *ε_rS_* matrix form is consistent with that of the *ε_rT_* matrix form as presented in Equation (8):

(8) $$\varepsilon_{rs}=\left[\begin{array}{ccc} \varepsilon_{rs11} & 0 & 0\\ 0 & 0 & 0\\ 0 & 0 & \varepsilon_{rs33} \end{array}\right]$$

The relationship between *ε_rT_* and *ε_rS_* is shown in Equation (9):

(9) $$\varepsilon_{rT}-\varepsilon_{rS}=de^{t}$$

Introducing the strain coefficient matrix *d* can better explain the deformation principle of the PM’s stack which is composed of multiple basic PM units. The advantages of the piezoelectric stack are long stroke and high displacement accuracy. The basic movement of the actuation leg is realized by *d*_33_ mode deformation in domain 2.1 and *d*_15_ mode deformation in domain 2.2.

**Figure 5:**
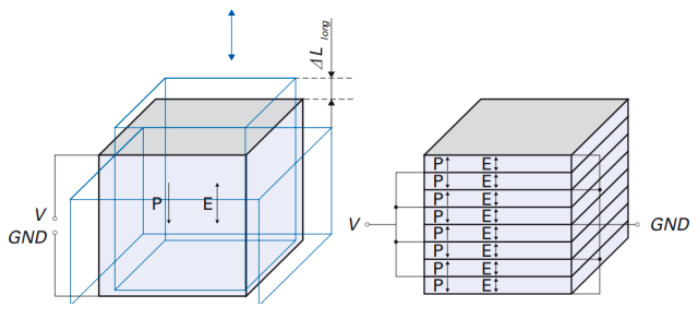
The principle of longitudinal deformation caused by the *d*_33_ mode.

When the piezoelectric stack consists of *n* basic PMs, and the electric field direction is the same as the polarization direction which is shown in Figure 5 [21], the *d*_33_ mode longitudinal deformation in domain 2.1 is expressed as:

(10) $$\Delta L=nd_{33}V=d_{33}nV,$$

**Figure 6:**
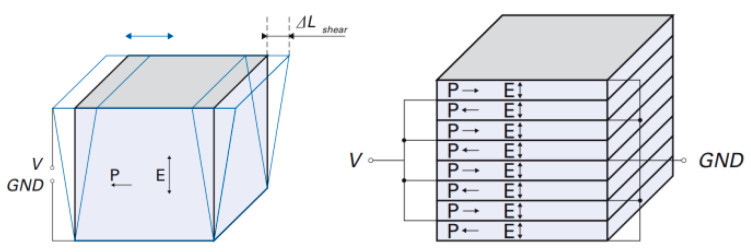
The principle of shear deformation caused by the *d*_15_ mode.

If the electric field direction is perpendicular to the polarization direction which is seen in Figure 6 [21], the *d*_15_ mode shear deformation in domain 2.2 is expressed as:

(11) $$\Delta L=nd_{15}V=d_{15}nV,$$

Assuming that the deformation degree of a basic PM unit is large enough, according to Equations (10) and (11), the deformation effect of *n* pieces of PM that are loaded with *V* volts is equivalent to that of a block of PM that is loaded with *nV* volts.

**Figure 7:**
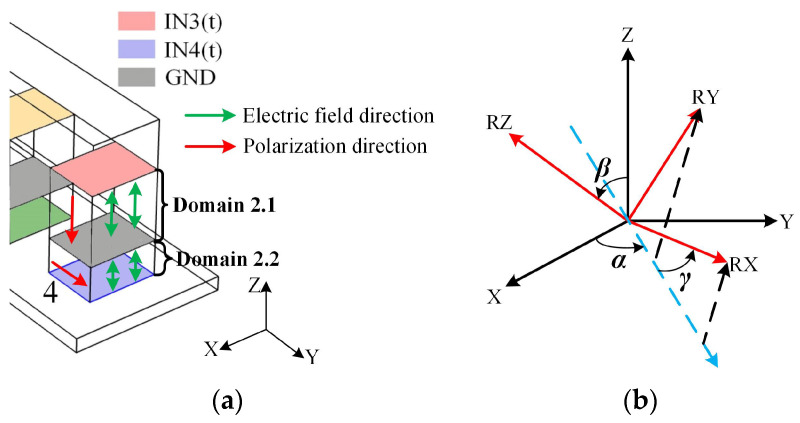
(**a**) Relationship between polarization direction and electric field direction; (**b**) relationship between rotating coordinate system and global coordinate system.

Supposing that the PIM simulation model is established in a global coordinate system (*X, Y, Z*), in general, the polarization direction of the PM defaults to the *z*-axis, so the *d*_33_ mode deformation in domain 2.1 is realized by applying an electric field in the *z*-axis, as presented in Figure 7a. But for the *d*_15_ mode deformation in domain 2.2, a rotation coordinate system (*RX, RY, RZ*) is configured to domain 2.2, making the polarization direction perpendicular to the electric field direction. The definition of the Euler angles *α*, *β*, *γ* are shown in Figure 7b. Equation (12) shows the relationship between the two coordinate systems:

(12) $$\left[\begin{array}{c} RX\\ RY\\ RZ \end{array}\right]=\left[\begin{array}{ccc} \cos\gamma & -\sin\gamma & 0\\ \sin\gamma & \cos\gamma & 0\\ 0 & 0 & 1 \end{array}\right]\left[\begin{array}{ccc} \cos\beta & 0 & \sin\beta \\ 0 & 1 & 0\\ -\sin\beta & 0 & \cos\beta \end{array}\right]\left[\begin{array}{ccc} 1 & 0 & 0\\ 0 & \cos\alpha & -\sin\alpha \\ 0 & \sin\alpha & \cos\alpha \end{array}\right]\left[\begin{array}{c} X\\ Y\\ Z \end{array}\right]$$

According to the polarization direction of domain 2.2 in Figure 7a, the three Euler angles are:

(13) $$\left[\begin{array}{ccc} \alpha & \beta & \gamma \end{array}\right]=\left[\begin{array}{ccc} 0 & \pi /2 & 0 \end{array}\right]$$

In summary, the form of all coefficient matrices in two constitutive equations must be in strict conformity with the above requirements to ensure the model convergence and correct deformation mode. Besides, a rotation coordinate system must be configured in the domain 2.2 to change the polarization direction.

#### 3.1.2. Nonlinear Contact Friction in Solid Mechanics

The contact friction among the four actuation feet and the driving shaft is analyzed by a Coulomb model. Consequently, the tangential traction is obtained from Equation (14), namely, PIM driving force.

(14) $$\begin{array}{l} T_{t}\leq \min(f+T_{cohe},T_{t.\max});f=\mu T_{n}\\ \vec{T_{t}}=\min(\frac{T_{t.crit}}{\left\|{\vec{T}}_{t.trial}\right\|},1){\vec{T}}_{t.trial}\\ {\vec{T}}_{t.trial}=-\rho_{t}g_{t};T_{n}=-\rho_{n}g_{n} \end{array}$$

where *T_t_* is the tangential traction; *f* is the sliding friction; *μ* is the coefficient of friction; *T_n_* is the normal force; *T_cohe_* is the cohesion sliding resistance; *T_t.max_* is the maximum tangential traction; *T_t.crit_* is the critical friction force, *T_t.trial_* is the trial tangential traction; *ρ_t_* is the penalty term used to express the relationship between the tangential contact force and the penetrations along the tangential direction, it is equivalent to the tangential stiffness; *g_t_* is the penetration along the tangential direction, which is equivalent to the tangential volume deformation. *ρ_n_* is the penalty term used to express the relationship between the normal contact force and the penetrations along the normal direction; it is equivalent to the normal stiffness; *g_n_* is the penetration along the normal direction which is equivalent to the normal volume deformation. Moreover, the model nonlinearity is reflected in: the contact interface is unknown in advance, including the area size, mutual position and contact state of the contact surface. They change with time (*ρ_t_*, *g_t_*, *ρ_n_*, *g_n_*), which needs to be determined in the solution process. In addition, the contact condition is a unilateral inequality constraint.

The increase of nonlinear components makes the convergence of the model very difficult. It is possible to solve the convergence problem and determine contact traction and friction force by setting the maximum number of iterations in the model solver.

#### 3.1.3. Electrostatics in Piezoelectric Materials

The electrostatic physical field is applied to domains 2.1 and 2.2. The entire physical process can be described by Equation (15):

(15) $$\begin{array}{l} -\nabla V=E\\ \varepsilon_{0}\varepsilon_{r}E+P=D\\ \nabla \cdot D=\rho \end{array}$$

where *V* is the input voltage signal, *P* is the polarization vector field, *ρ* is the space charge density, and *ε_r_* is the relative permittivity matrix; it could be *ε_rT_* or *ε_rS_*, depending on the form of the piezoelectric constitutive equation used. Both *E* and *D* can be solved by Equation (15a) and (15b):

(15a) −∇V=E,

(15b) $$\varepsilon_{0}\varepsilon_{r}E+P=D,$$

they are also the input information needed by the piezoelectric constitutive Equations (1) and (5) to solve the strain and stress. Therefore, the main function of electrostatics is to provide the excitation input in the system model.

### 3.2. Setting of Material, Excitation and Mesh

In this section, the material properties of domain 1, domains 2.1 and 2.2, and domain 3 are introduced, respectively. Next, the input excitation is set to achieve the PIM movement. Finally, the model mesh is described.

#### 3.2.1. Setting of Key Piezoelectric Materials and Others

Based on the above theory analysis, the coefficient characteristics matrices of PIC255 in domain 2.1 and domain 2.2 are the key information that must be determined, for example, *S_E_*, *d*, *ε_rT_* in the strain–charge equation and *c_E_*, *e*, *ε_rS_* in the stress–charge equation.

The coefficient matrices, *S_E_*, *d, ε_rT_* in the strain–charge are shown in Table 1.

The coefficient matrices, *c_E_*, *e*, *ε_rS_* in the stress–charge equation are shown in Table 2. The coefficient matrix form is determined by the crystal structure of the PIC255 and polarization. The incorrect coefficient matrix form will cause the model to not be solved, even if the model is successfully solved under certain circumstances, the deformation characteristics of the PM may be incorrect.

As for the driving shaft in the domain 1 and fixed axle in the domain 3, C1045 is the driving shaft material according to Reference [24]. Steel AISI 4340 is applied to the fixed axle, and the high rigidity can meet the requirements. Table 3 shows the basic characteristics of the three materials, including PIC255.

#### 3.2.2. Setting of Input Excitation

The laws of motion of the PIM can be obtained from Reference [12] and summarized below:

1. The movement state of the non-adjacent actuation legs is the same;
2. For the adjacent actuation legs, when one leg has completed all movements in one cycle, the other leg starts to follow same movements.

**Figure 8:**
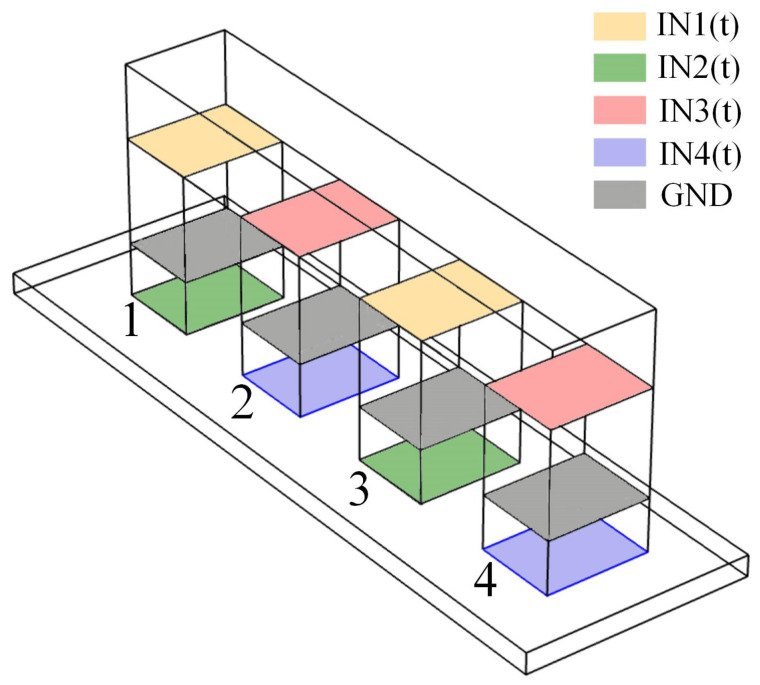
The distribution of input excitation.

Figure 8 shows the excitation distribution of the four actuation legs (NO. 1, NO. 2, NO. 3, and NO. 4) in the model. According to the first law of motion, the movement gesture of NO.1 is the same as that of NO. 3, and NO. 2 and NO. 4 also follow this law of motion. According to the second law of motion, when NO. 1 and NO. 3 have completed all movement gesture in a cycle, NO. 2 and NO. 4 start to follow the same movement gesture in the next cycle.

The four types of excitation functions are listed below:

(16) $$\begin{array}{cc} IN1(t)=\{\begin{array}{cc} -m_{0}AU & t\in \left[0,T/2\right]\\ m_{0}(8Ut/T-5U) & t\in (T/2,3T/4]\\ m_{0}(-8Ut/T+7U) & t\in (3T/4,T] \end{array} & IN2(t)=\{\begin{array}{ll} m_{1}(-4Ut/T+U) & t\in \left[0,T/2\right]\\ m_{1}(4Ut/T-3U) & t\in (T/2,T] \end{array}\\ IN3(t)=\{\begin{array}{ll} m_{0}(8Ut/T-U) & t\in \left[0,T/4\right]\\ m_{0}(-8Ut/T+3U) & t\in (T/4,T/2]\\ -m_{0}Ut & \in (T/2,T] \end{array} & IN4(t)=\{\begin{array}{ll} m_{1}(4Ut/T-U) & t\in \left[0,T/2\right]\\ m_{1}(-4Ut/T+3U) & t\in (T/2,T] \end{array}, \end{array}$$

where *m*_0_ and *m*_1_ represent the magnification factor of the longitudinal and shear deformation respectively, depending on number of layers of piezoelectric stack. There, these two magnification factors correspond to *n* in Equations (10) and (11). The *m*_0_ and *m*_1_ of P-123.01, P-143.01, P-153.01 are presented in Table 4. *U* is the initial voltage, corresponding to *V* in the Equation (15). *T* is the time that requires to complete all movement gesture of one actuation leg. The excitation function needs to be smooth to prevent the model from non-convergence when the magnification factors or initial voltage is set large. If the smooth effect is not good, the smooth transition area needs to be enlarged. According to the official recommended voltage of PIC255, *U* is set to 250 volts. Moreover, *T* is one second, the solver time step should be set depending on the model convergence, and the recommended step size is 1 × 10^−2^ to 1 × 10^−5^. 

#### 3.2.3. Setting of Model Mesh

**Figure 9:**
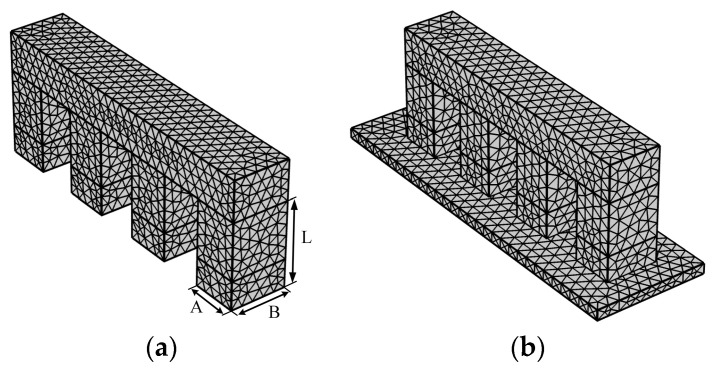
Model mesh: (**a**) model without driving shaft; (**b**) the full model.

In response to coupling and nonlinear problems, a fine mesh was utilized to divide the model, but it can bring about a sharp increase in the degree of solution freedom. The computer memory and solution time were greatly increased which seriously reduced the efficiency of model debugging. Therefore, the principle of mesh generation is to minimize the number of meshes without affecting model solution. Figure 9 shows the mesh of the model without driving shaft and the full model. The free tetrahedral mesh was used to partition the two model with super refinement level mesh. In Figure 9a, A is the length of the actuation leg, B and L are the width and height of leg separately. The model with driving shaft is shown in Figure 9b, the mesh size range of P123.01 (A = B = 5 mm, L = 7.5 mm) is 0.0795 mm–1.86 mm, the solution freedom is 45,610. If the extremely fine-level mesh is adopted, the solution freedom becomes 205,564, resulting in too long of a solution time. As for P143.01 (A = B = 10 mm, L = 7.5 mm) and P153.01 (A = B = 16 mm, L = 15.5 mm), the mesh size range for both of them were 0.012–1.2 mm and 0.024–1.8 mm, the solution freedoms were 150,148 and 172,651 respectively. The critical equations, including piezoelectric constitutive equations (Equations (1) or (5)), Coulomb friction model (Equation (14)), electrostatics (Equation (15)), were solved in the proposed mesh system.

## 4. Results and Discussion

The realization of the PIM motion gesture determined the feasibility of the multi-physics field simulation model of PIM. The motion gesture (*d*_33_ mode and *d*_15_ mode), driving force, and travel of the PIM were the key factors in the PIM design. All of the above were closely related to the material properties and size (A, B, L) of the PIC255.

### 4.1. Gesture Analysis of the Four Actuation Legs

**Figure 10:**
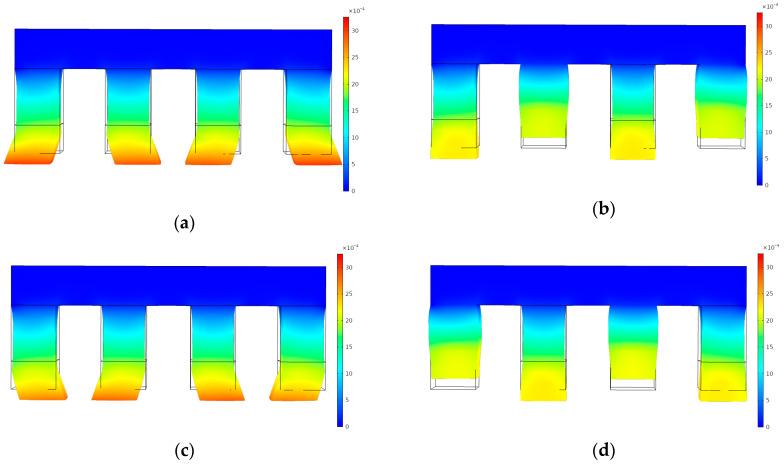

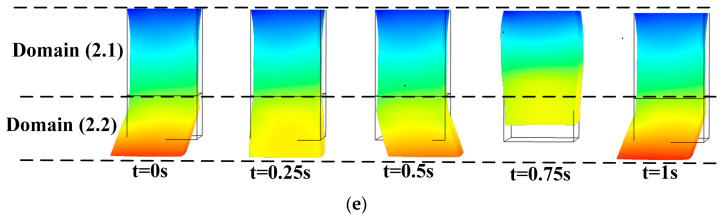
The gesture change of the actuation leg: (**a**) *t* = 0 s; (**b**) *t* = 0.25s; (**c**) *t* = 0.5 s; (**d**) *t* = 0.75 s; (**e**) one-leg gesture change in one second.

The longitudinal and shear deformation that was caused by the *d*_33_ and *d*_15_ mode were crucial to the realization of the inchworm gesture. Figure 10 shows the movement gesture change of the actuation legs in the inchworm model without a driving shaft. The gesture changes of four actuation legs are presented in Figure 10a–d; in addition, the leg gesture at 0 s was exactly the same as the gesture at 1 s. It can be concluded that the movements of non-adjacent actuation legs are the same at each time point. For a more detailed analysis, one-leg gesture change is highlighted in Figure 10e, as for adjacent actuation legs, when the “walk” action of one actuation ends, the other one starts to “walk”. From 0 s to 0.5 s, the domain 2.1 was always in the elongation state, and domain 2.2 had shear deformation from left to right. In 0.5–0.75 s, the domain 2.1 contracted upward. Then it extended downward from 0.75 to 1 s. As for the domain 2.2, the domain 2.2 deformed laterally from right to left in 0.5–1 s. The motion gesture of the actuation leg was consistent with the video provided by PI motion in Reference [22]. The simulation animation of P-123.01, P-143.01, and P-153.01 is shown in the Supplementary Materials.

### 4.2. Driving Force Analysis

The driving force was equal to the contact tangential traction. If the driving force is greater than the maximum shear load (MSL), the piezoelectric stack will be broken in the shear direction. The maximum driving force was equal to the maximum shear load.

Table 5 presents the standard size and MSL of P-123.01, P-143.01, and P-153.01 which were obtained from PI Motion.

**Figure 11:**
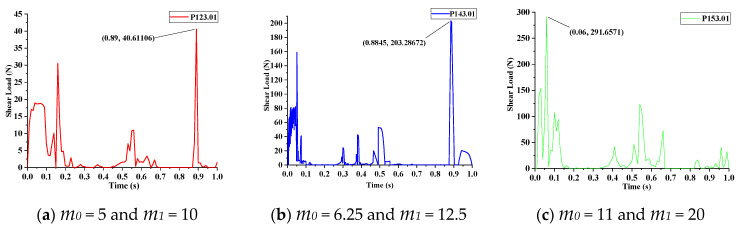
MSL of three shear actuation products: (**a**) P-123.01; (**b**) P-143.01; (**c**) P-153.01.

Equation (17) is the absolute error formula. Figure 11 depicts the contact tangential traction of the above three products over time in the simulation, the MSL of P-123.01, P-143.01, and P-153.01 were 40.611N, 203.286N, and 291.657N respectively, the corresponding absolute error were 1.50%, 1.65%, and 2.78% and which are shown in Table 5. Therefore, the maximum shear load increases with the increase of PM size; this conclusion is also consistent with the MSL, as shown in Table 5.

(17) $$\mathrm{Absolute}\;\mathrm{Error}=\frac{\left|\mathrm{Simulation}\;\mathrm{value}\;-\;\mathrm{Standard}\;\mathrm{value}\right|}{\mathrm{Standard}\;\mathrm{value}}.$$

### 4.3. Travel Analysis

**Figure 12:**
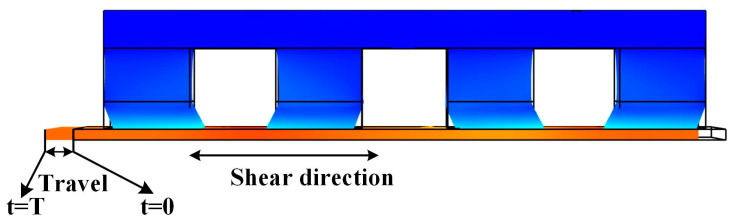
Schematic diagram of PIM travel.

**Figure 13:**
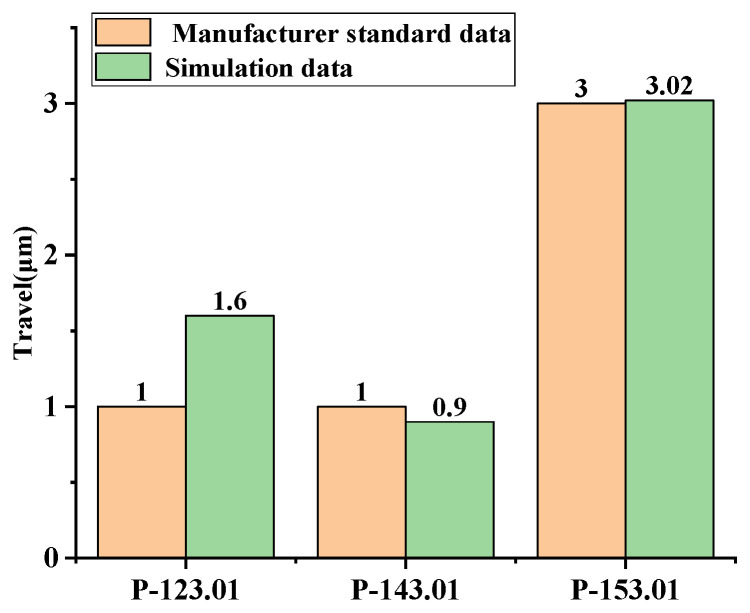
Travel data for the three products from the manufacturer and the simulation.

The travel is the displacement of the driving shaft in the shear direction during the action completed period (*T*) as illustrated in Figure 12. The travel data from manufacturer and simulation are compared in Figure 13, P-123.01′s travel in simulation was 1.6μm, and there was an error of 0.6 μm compared with the manufacturer’s standard data. The travel of P-143.01 and P-153.01 were 0.9 μm and 3.02 μm, and the errors were 0.1 μm and 0.02 μm, respectively. In addition, a large number of meshes were needed for high-solution accuracy. Model calculations with many meshes and the coupling of multiple physical fields have high requirements for computer performance and need a long solution time. 

## 5. Conclusions

In this paper, a multi-physics coupling model of an inchworm motor was established based on PI Motion’s advanced inchworm motor products. The motion gesture and performance parameters of the inchworm motor were the research priority for the motor design and performance estimation. Three different sizes of products, including P123.01, P143.01, P153.01, were simulated with 250 V initial voltage in one second. The key piezoelectric material was PIC255. Two conclusions are as follows:

The *d*_33_ and *d*_15_ mode deformations of PIC255 were perfectly realized by simulation. That is, the inchworm’s gesture characteristic was perfectly realized, and it is highly consistent with PI Motion’s products. The realization of motion gesture proves that the multi physical field coupling simulation is feasible for assisting the PIM design.

The maximum driving force error of P123.01 between the simulation data and the official data was 2.8%, the errors for P143.01 and P153.01 were 1.50% and 1.65%, respectively. The driving forces of the three types of products obtained from the simulation were close to those provided by the actual official data. In addition, the driving force increases with the increasing piezoelectric material size. As for the PIM travel, there was a maximum travel error of 0.6 μm between the simulation and the official standard for the P123.01, the errors for P143.01 and P153.01 were 0.1 μm and 0.02 μm. The travel data from the simulation and the official standard was well matched. So, the performance of the PIM can be directly obtained by multi-physics field coupling simulation.

In conclusion, the multi-physics field coupling simulation model involving solid mechanics and electrostatics is suitable for PIM performance evaluation and assisting motor design.

## Figures and Tables

**Table 1:** The Coefficient summary of Strain-Charge form.

Flexibility Coefficient	Value (m^2^/N)	Strain Coefficient	Value (m/V)	Dielectric Constant	Value
*S*_E11_	1.606 × 10^−11^	*d*_33_	3.996 × 10^−10^	ε*_rT_*_11_	1852
*S*_E12_	−5.685 × 10^−12^	*d*_15_	6.174 × 10^−10^	ε*_rT_*_33_	1751
*S*_E13_	−7.454 × 10^-12^	*d*_31_	−1.867 × 10^-10^		
*S*_E33_	1.909 × 10^−11^				
*S*_E44_	4.699 × 10^−11^				
*S*_E66_	4.350 × 10^−11^				

**Table 2:** The Coefficient summary of the stress–charge form.

Stiffness Coefficient	Value (N/m^2^)	Strain Coefficient	Value (N/Vm)	Dielectric Constant	Value
*c_E_*_11_	1.327 × 10^11^	*e*_33_	15.68	ε*_rS_*_11_	936
*c_E_*_12_	8.667 × 10^10^	*e*_15_	13.14	ε*_rS_*_33_	759
*c_E_*_13_	8.563 × 10^10^	*e*_31_	−6.73		
*c_E_*_33_	1.192 × 10^11^				
*c_E_*_44_	2.128 × 10^10^				
*c_E_*_66_	2.299 × 10^10^				

**Table 3:** The basic characteristics of C1045 and Steel AISI 4340.

Type	Density (kg/m^3^)	Young’s Modulus	Poisson’s Ratio (GPa)
C1045	7890	200	0.3
Steel AISI 4340	7850	205	0.35
PIC255	7800	-	0.35

**Table 4:** The magnification factors of three products.

Type	*m*_0_	*m*_1_
P-123.01	5	10
P-143.01	6.25	12.5
P-153.01	11	20

**Table 5:** Product information and error comparison.

Type	A × B × L (mm)	MSL (N)	Absolute Error (%)
P-123.01	5 × 5 × 7.5	40	1.50%
P-143.01	10 × 10 × 7.5	200	1.65%
P-153.01	16 × 16 × 15.5	300	2.78%

## Supplementary Materials

The following are available online at https://www.mdpi.com/1996-1944/13/18/3976/s1, Video S1–S6: Simulation.
