# Supplementary figures and images for: Modeling of Motion Characteristics and Performance Analysis of an Ultra-Precision Piezoelectric Inchworm Motor
Source: Materials (Basel). 2020 Sep 8;13(18):3976. doi: 10.3390/ma13183976 (PMC7557391; doi:10.3390/ma13183976)

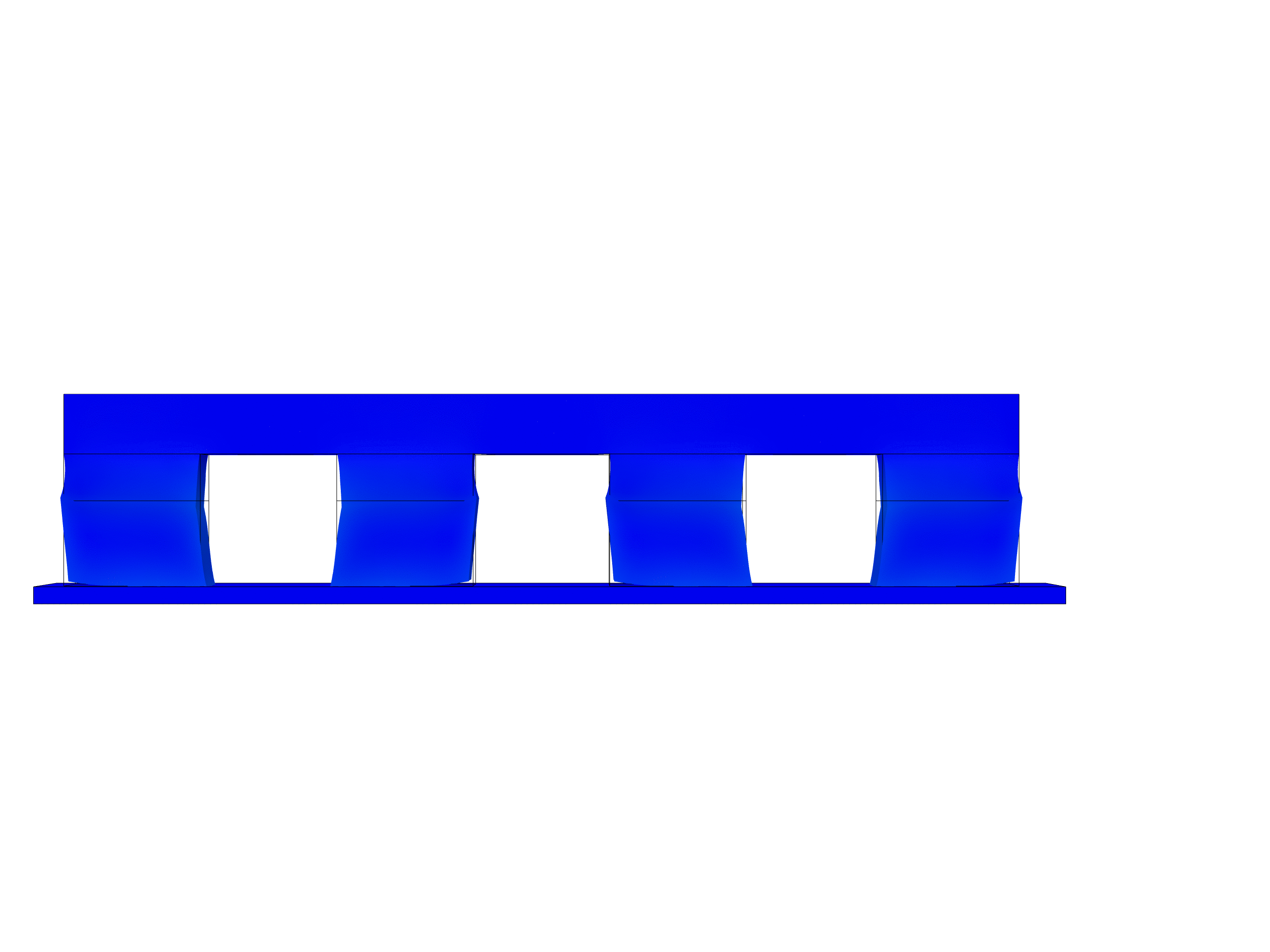

Supplement: Supplementary file 1 [file materials-13-03976-s001.zip › P153.01/10FPS.gif]

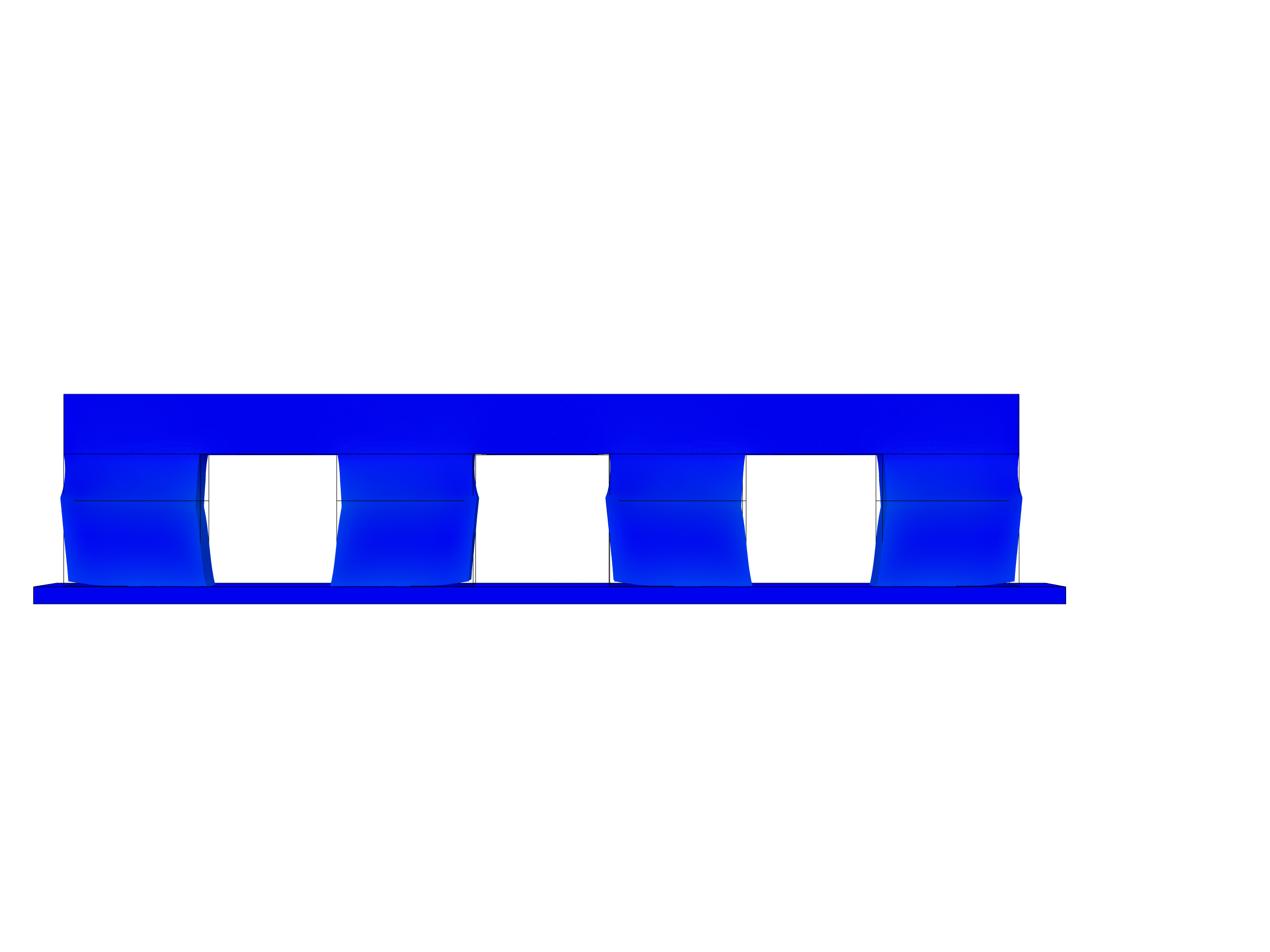

Supplement: Supplementary file 1 [file materials-13-03976-s001.zip › P153.01/5FPS.gif]

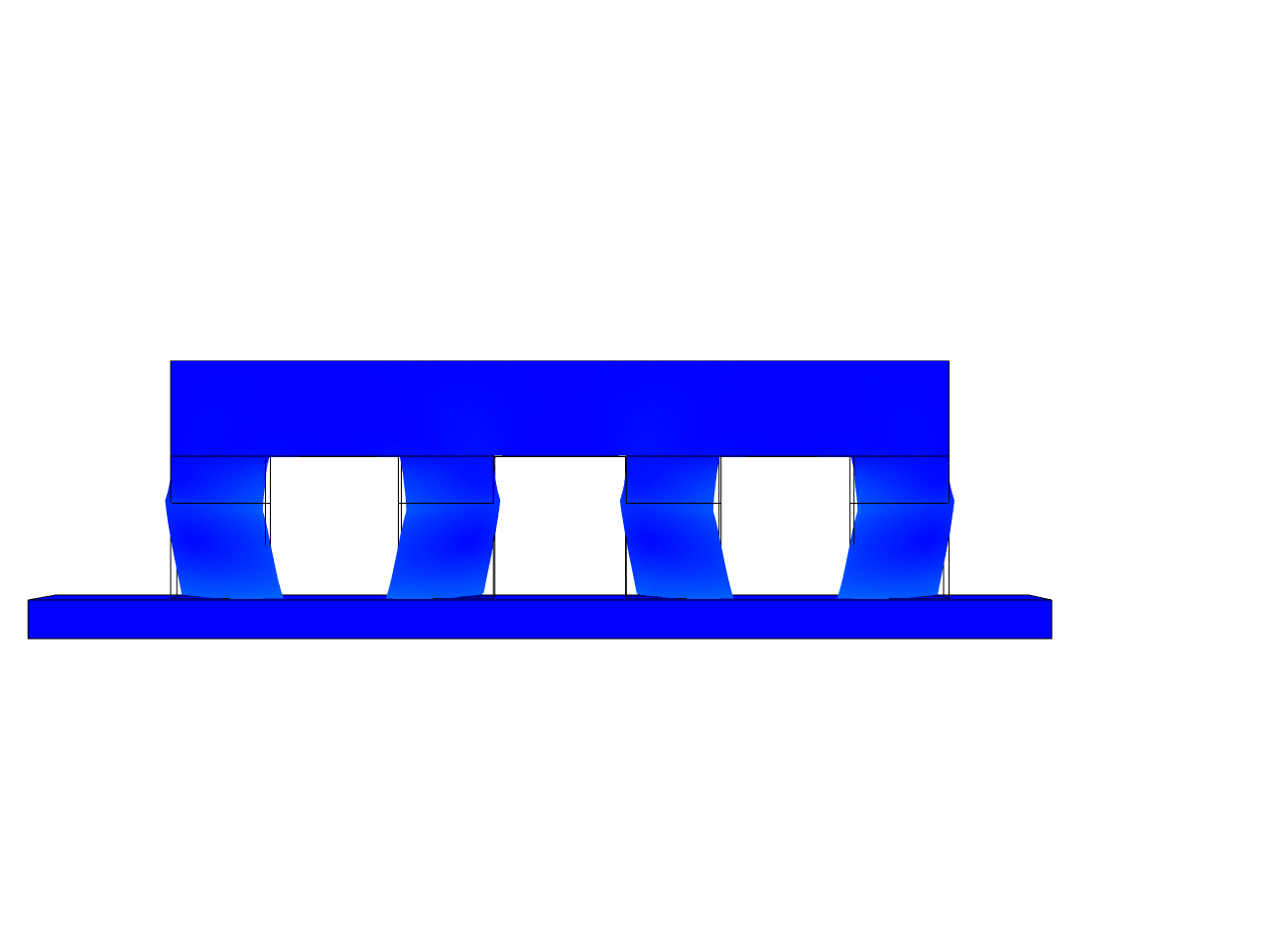

Supplement: Supplementary file 1 [file materials-13-03976-s001.zip › P123.01/10 FPS.gif]

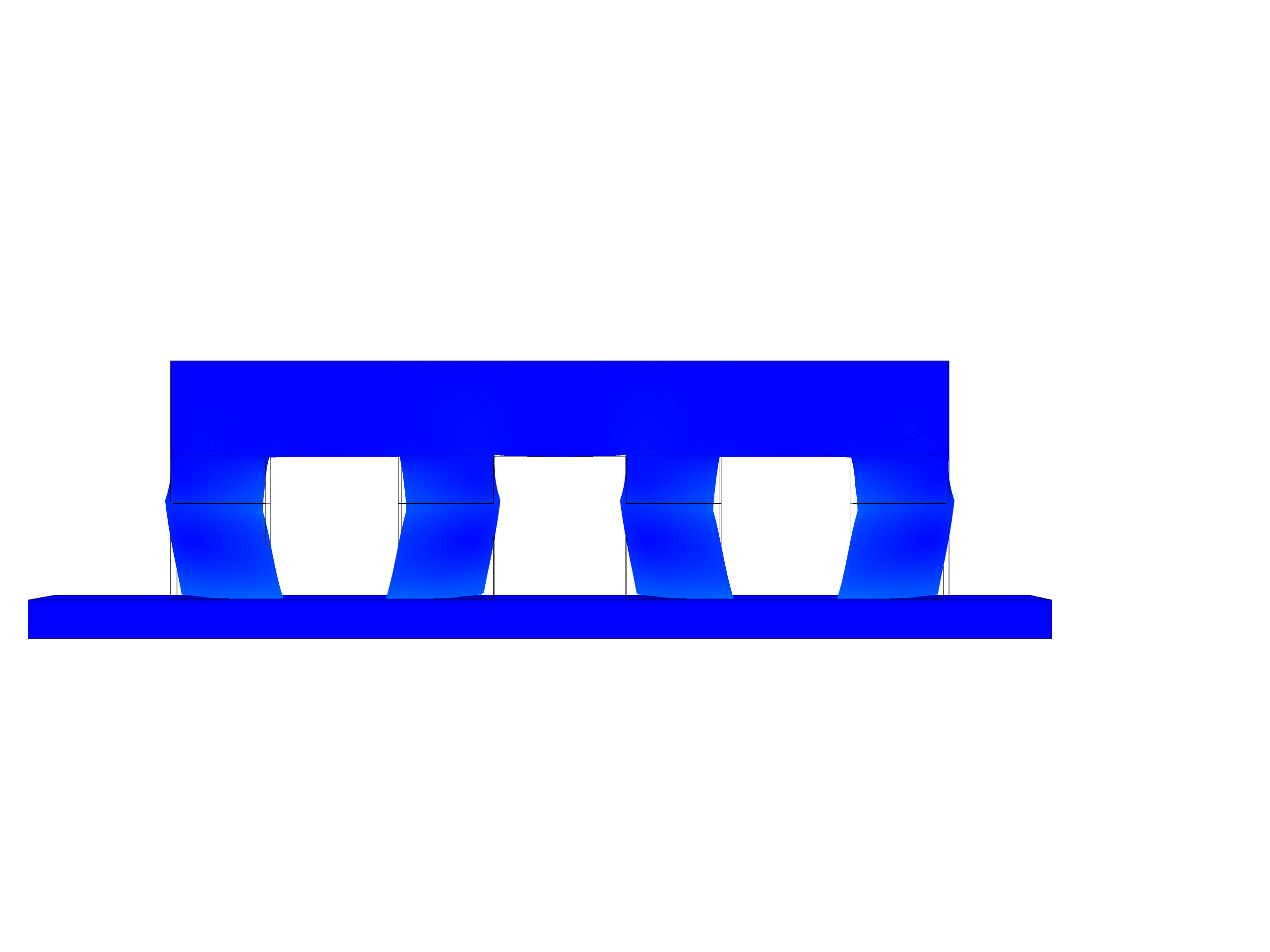

Supplement: Supplementary file 1 [file materials-13-03976-s001.zip › P123.01/5 FPS.gif]

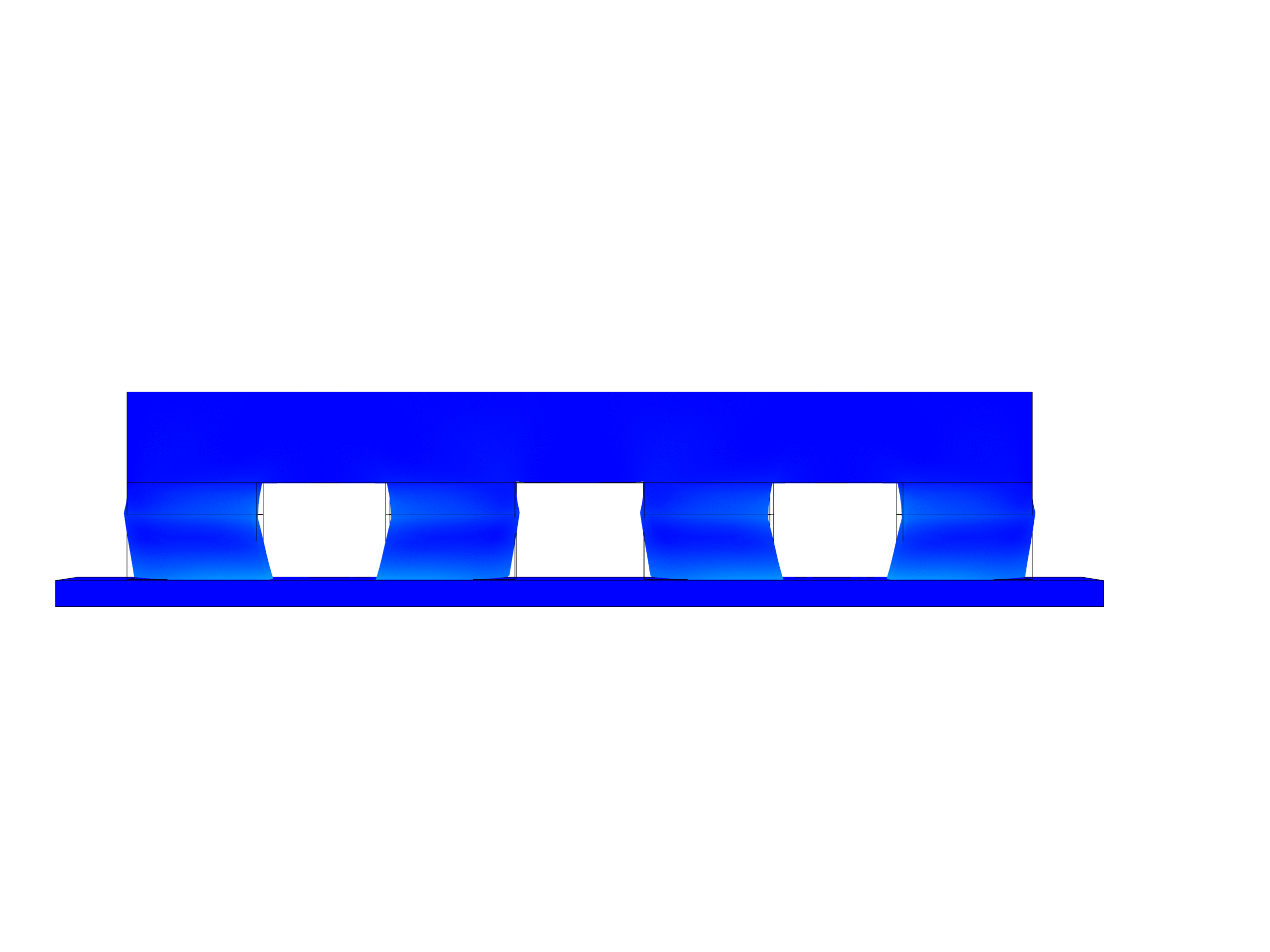

Supplement: Supplementary file 1 [file materials-13-03976-s001.zip › P143.01/10FPS.gif]

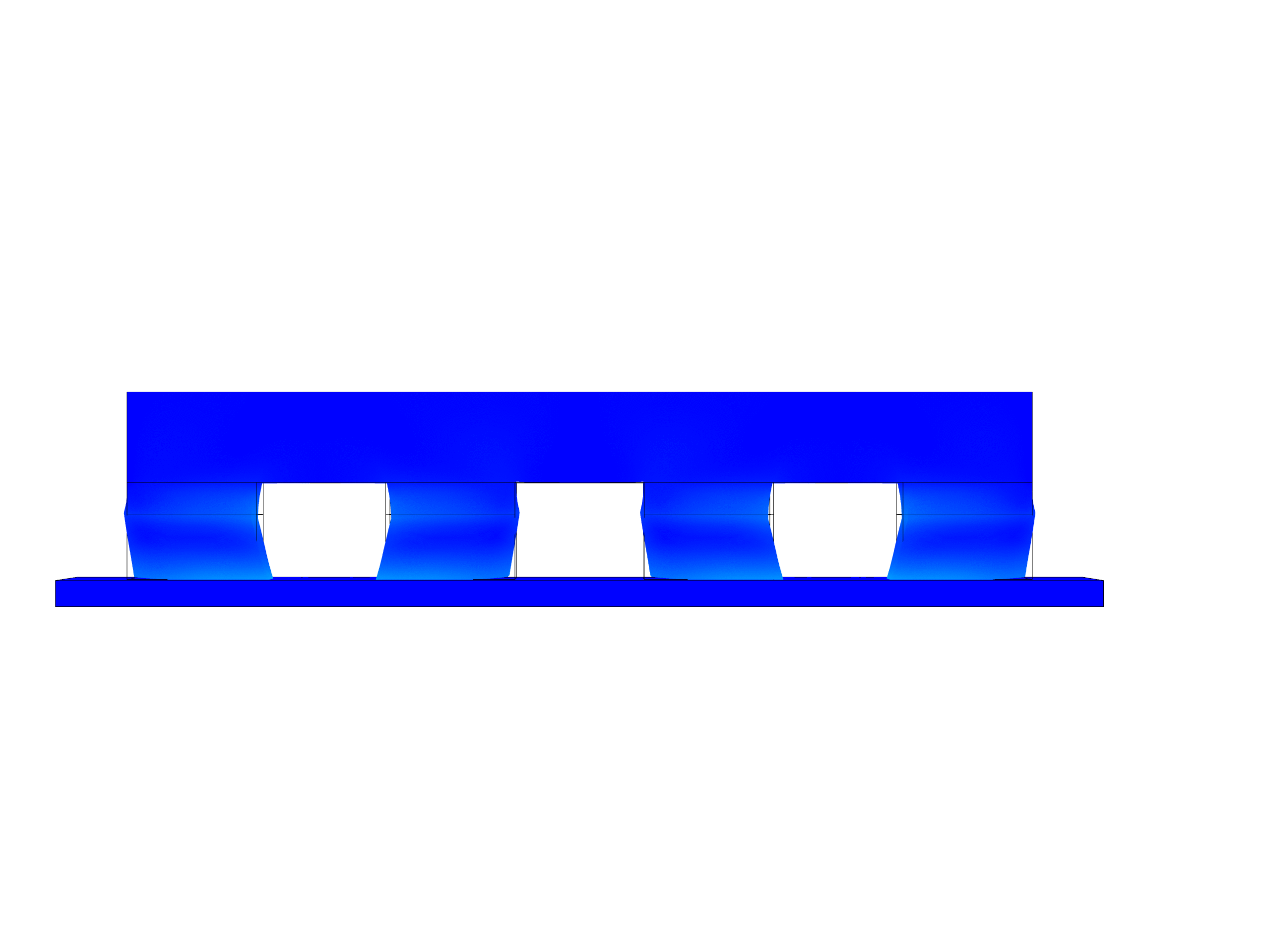

Supplement: Supplementary file 1 [file materials-13-03976-s001.zip › P143.01/5FPS.gif]
